# Inflammation and Mechanical Stress Stimulate Osteogenic Differentiation of Human Aortic Valve Interstitial Cells

**DOI:** 10.3389/fphys.2018.01635

**Published:** 2018-11-20

**Authors:** Maria Bogdanova, Aleksandra Kostina, Katarina Zihlavnikova Enayati, Arsenii Zabirnyk, Anna Malashicheva, Kåre-Olav Stensløkken, Gareth John Sullivan, Mari-Liis Kaljusto, John-Peder Escobar Kvitting, Anna Kostareva, Jarle Vaage, Arkady Rutkovskiy

**Affiliations:** ^1^Department of Molecular Medicine, Institute of Basic Medical Sciences, University of Oslo, Oslo, Norway; ^2^Almazov National Medical Research Centre, St. Petersburg State University, St. Petersburg, Russia; ^3^ITMO University, Institute of Translational Medicine, St. Petersburg, Russia; ^4^Faculty of Biology, St. Petersburg State University, St. Petersburg, Russia; ^5^Norwegian Center for Stem Cell Research, Oslo University Hospital and University of Oslo, Oslo, Norway; ^6^Institute of Immunology, Oslo University Hospital, Oslo, Norway; ^7^Hybrid Technology Hub-Centre of Excellence, Institute of Basic Medical Sciences, University of Oslo, Oslo, Norway; ^8^Department of Cardiothoracic Surgery, Oslo University Hospital, Oslo, Norway; ^9^Department of Woman and Children Health, Karolinska Institutet, Stockholm, Sweden; ^10^Department of Emergency Medicine and Intensive Care, Oslo University Hospital, Oslo, Norway; ^11^Institute of Clinical Medicine, University of Oslo, Oslo, Norway; ^12^Department of Cardiology, Akershus University Hospital, Oslo, Norway

**Keywords:** inflammation, mechanical stress, valve interstitial cells, valve calcification, osteogenic differentiation, extracellular matrix

## Abstract

**Background:** Aortic valve calcification is an active proliferative process, where interstitial cells of the valve transform into either myofibroblasts or osteoblast-like cells causing valve deformation, thickening of cusps and finally stenosis. This process may be triggered by several factors including inflammation, mechanical stress or interaction of cells with certain components of extracellular matrix. The matrix is different on the two sides of the valve leaflets. We hypothesize that inflammation and mechanical stress stimulate osteogenic differentiation of human aortic valve interstitial cells (VICs) and this may depend on the side of the leaflet.

**Methods:** Interstitial cells isolated from healthy and calcified human aortic valves were cultured on collagen or elastin coated plates with flexible bottoms, simulating the matrix on the aortic and ventricular side of the valve leaflets, respectively. The cells were subjected to 10% stretch at 1 Hz (FlexCell bioreactor) or treated with 0.1 μg/ml lipopolysaccharide, or both during 24 h. Gene expression of myofibroblast- and osteoblast-specific genes was analyzed by qPCR. VICs cultured in presence of osteogenic medium together with lipopolysaccharide, 10% stretch or both for 14 days were stained for calcification using Alizarin Red.

**Results:** Treatment with lipopolysaccharide increased expression of osteogenic gene bone morphogenetic protein 2 *(BMP2)* (5-fold increase from control; *p* = 0.02) and decreased expression of mRNA of myofibroblastic markers: α-smooth muscle actin *(ACTA2)* (50% reduction from control; *p* = 0.0006) and calponin *(CNN1)* (80% reduction from control; *p* = 0.0001) when cells from calcified valves were cultured on collagen, but not on elastin. Mechanical stretch of VICs cultured on collagen augmented the effect of lipopolysaccharide. Expression of periostin *(POSTN)* was inhibited in cells from calcified donors after treatment with lipopolysaccharide on collagen (70% reduction from control, *p* = 0.001), but not on elastin. Lipopolysaccharide and stretch both enhanced the pro-calcific effect of osteogenic medium, further increasing the effect when combined for cells cultured on collagen, but not on elastin.

**Conclusion:** Inflammation and mechanical stress trigger expression of osteogenic genes in VICs in a side-specific manner, while inhibiting the myofibroblastic pathway. Stretch and lipopolysaccharide synergistically increase calcification.

## Introduction

Aortic valve calcification is the most common valve disease in the developed countries (Beckmann et al., 2010), believed to be caused by changes in the cell biology and phenotype of valve interstitial cells (VICs) (Leopold, 2012; Rutkovskiy et al., 2017). Because the occurrence of heart valve calcification will increase in an aging population, it is imperative to find treatments less invasive than heart surgery and more effective than transcatheter valve replacement. Elucidating the cellular and molecular mechanisms of valve calcification may open up for new pharmacological therapies (Mathieu et al., 2014). Due to a lack of good animal models studying VICs in culture is the best model alternative (Bowler and Merryman, 2015). VICs in healthy aortic valves have most characteristics of quiescent fibroblasts while in calcified valves their phenotype starts to resemble either myofibroblasts or osteoblasts (Miller et al., 2011; Rajamannan et al., 2011). Inflammation is believed to be one of the key mechanisms of aortic valve calcification (Mohler et al., 2001; Coté et al., 2013; Pawade et al., 2015; Rutkovskiy et al., 2017) Moreover, bacteria have been found in valve cusps on autopsy (Higuchi Mde et al., 2002), suggesting the possibility of a direct pathogenic effect of microbes on cell differentiation. Lipopolysaccharide (LPS) is an endotoxin of gram-negative bacteria interacting with toll-like receptors 2 and 4 which are expressed on the plasmalemma of human VICs (Meng et al., 2008; Yang et al., 2009a; López et al., 2012). Stimulation of VICs with LPS promotes expression of osteogenic markers, such as bone morphogenetic protein 2 (BMP2), alkaline phosphatase (ALP) (Babu et al., 2008; Meng et al., 2008; Yang et al., 2009a; Wang et al., 2014; Zeng et al., 2014) and runt-related transcription factor 2 (RUNX2) (Meng et al., 2008), mediated by pro-inflammatory mediators, such as intracellular adhesion molecule 1 (ICAM1) (Meng et al., 2008; Song et al., 2011; Wang et al., 2014).

Aortic valves leaflets are constantly subjected to mechanical stress (Bäck et al., 2013) and mechanical stress exceeding the physiological range may cause calcification (Lehmann et al., 2009; Rutkovskiy et al., 2017). *Ex vivo* cyclic stretch of human VICs has already been found to trigger expression of osteogenic markers, such as BMP2 (Balachandran et al., 2010; Ferdous et al., 2013), RUNX2 (Balachandran et al., 2010) and some others (Lehmann et al., 2009). However, its effect has not been compared or combined with inflammation.

In calcified aortic valve leaflets, calcium deposits are solely observed on the aortic side (Yip and Simmons, 2011). The reasons for this are unknown, but may be caused by different mechanical and biochemical stimuli including different composition of extracellular matrix (Chen and Simmons, 2011; Hinton and Yutzey, 2011; Yip and Simmons, 2011). The matrix of the *fibrosa* layer (the aortic side) is primarily composed of collagen, whereas the *ventricularis* layer (ventricular side) contains high concentrations of elastin (Latif et al., 2005; Yip and Simmons, 2011).

The extracellular matrix may influence cellular processes by signaling through adhesion receptors, regulate the presentation of growth factors and cytokines to cells and transduce hemodynamic forces (Chen and Simmons, 2011). The possible role for extracellular matrix on the calcification process is poorly understood. VICs cultured on different types of coatings show different ability to calcify (Benton et al., 2008; Rodriguez and Masters, 2009; Yip et al., 2009; Rutkovskiy et al., 2017). However, collagen and elastin have not been compared head-to-head in their ability to modify response to pro-calcific stimuli.

The signaling pathways of calcification may also differ between VICs from healthy and calcified valves. Consequently, when stimulating VICs to investigate the cellular and molecular mechanisms of calcification, cells from both healthy and calcified valves ought to be studied. Therefore, we obtained both and subjected them to similar treatments. The aims of this study were: (1) to investigate the effect of inflammation and mechanical stress on induced osteogenic differentiation with calcification of cultured human VICs, either alone or in combination; (2) to compare the effect of inflammation and mechanical stress on osteogenic differentiation in VICs cultured on either collagen or elastin, representing the extracellular matrix on the aortic and ventricular sides of the leaflets, respectively; and (3) to investigate effects of inflammation and mechanical stress on VICs from healthy and calcified aortic valves.

## Materials and methods

Aortic valves were harvested at the Department of Cardiothoracic Surgery, Oslo University Hospital, Oslo, Norway. All procedures were approved by the local Ethical Committee and were performed in accordance with the principle of the Declaration of Helsinki. Healthy valve leaflets were collected from explanted hearts of heart transplant recipients without history of heart valve disease. Calcified aortic valves were harvested from patients with undergoing aortic valve replacement after written informed consent. All the valves included in the study had tricuspid morphology.

### Cell isolation and culture

The excised aortic valve leaflets were treated with 1 mg/mL of collagenase II (Worthington Biochemical Corporation, LS004177) made up in Dulbecco's Modified Eagle Medium (DMEM) (Life technologies, 41966-052) for 10 min at 37°C and endothelial cells were scraped off with cotton swabs from both sides. Then the leaflets were subjected to overnight digestion with collagenase II at 37°C. The cell suspension was then homogenized by repeated pipetting up and down, followed by centrifugation at 300 g for 5 min and cell pellet was collected. The VICs were cultured in cell culture medium containing DMEM (Gibco by Life technologies, 41966-052), 10% Fetal Bovine Serum (FBS, Gibco by Life Technologies, 10270-106) and Penicillin/Streptomycin (P/S, 1%, Gibco by Life technologies, P4333) at 37°C in 5% CO_2_ until confluence of 70–80% before passaging. The cells from passages 2 to 6 were used in the experiments. An overview of the experimental groups is shown in Table 1. Given *n* are biological replicates of cells from different donors. Control groups were from the same donors as the treatments in Series 1–4. The cells from the same donors at the same passage were used in the experiments on elastin and on collagen coatings in Series 2 and Series 4.

**Table 1:** Experimental groups.

**Series**	**Groups**	**Healthy**	**Calcified**	**Flex** **(10% stretch)**	**LPS** **(0.1 μg/ml)**	**OM** **(osteogenic medium)**
Series 1	Group 1.1	*n* = 3	_	_	_	_
	Group 1.2	*n* = 3	_	_	+	_
	Group 1.3	*n* = 3	_	_	_	+
	Group 1.4	*n* = 3	_	_	+	+
Series 2	Group 2.1	*n* = 6	_	+	_	_
	Group 2.2	*n* = 6	_	_	_	+
	Group 2.3	*n* = 6	_	_	+	+
	Group 2.4	*n* = 6	_	+	_	+
	Group 2.5	*n* = 6	_	+	+	+
Series 3	Group 3.1	*n* = 4	_	_	+	_
	Group 3.2	_	*n* = 4	_	+	_
Series 4	Group 4.1	*n* = 6	_	_	_	_
	Group 4.2	*n* = 6	_	_	+	_
	Group 4.3	*n* = 6	_	+	_	_
	Group 4.4	*n* = 6	_	+	+	_
	Group 4.5	_	*n* = 6	_	_	_
	Group 4.6	_	*n* = 6	_	+	_
	Group 4.7	_	*n* = 6	+	_	_
	Group 4.8	_	*n* = 6	+	+	_

### Measurement of calcification

In experiments studying the effect of LPS on calcification (Groups 1.1–1.4), VICs isolated from healthy aortic valves were seeded 25,000 per well in 24-well plates pre-coated with collagen I (Gibco, A10483-01) and stimulated with 0.1 μg/ml LPS (E. coli 0111:B4, Sigma, L4391) and/or osteogenic medium (cell culture medium supplemented with 50 μM ascorbic acid, 0.1 μM dexamethasone and 10 mM beta-glycerophosphate). As a control, VICs were cultured in cell culture medium without any stimulation at the same time. Medium in all groups was changed twice a week for 14 days.

For the quantification of calcification following treatment with stretch and LPS (Groups 2.1–2.5), VICs isolated from healthy aortic valves were seeded in concentration of 125,000 cells per well on type I collagen and elastin pre-coated BioFlex 6-well cell culture plates (Dunn Labortechnik, BF-3001C and BF-3001E). After 24 h the cells were stimulated with osteogenic medium alone or together with cyclic stretch and/or 0.1 μg/ml LPS (E. coli 0111:B4, Sigma, L4391). Cyclic stretch was applied to the culture plates at 1 Hz (0.5 s of 10% stretch alternating with 0.5 s of relaxation) by using a computer-controlled vacuum stretch bioreactor (FlexCell International Corporation, FX-5000T) inside the 5% CO_2_ incubator at 37°C. Cyclic stretch was applied for 2 h per day during the first 7 days of 2-weeks experiment. VICs stimulated with cyclic stretch alone without osteogenic medium in the same time frame were used as control. The medium in all treatment groups was changed twice a week.

Following 2 weeks of stimulation the cells were stained with Alizarin Red (Sigma-Aldrich, A5533) according to the manufacturer's protocol for calcification. Briefly, the VICs still attached to the matrix were washed with PBS and fixed with 70% ethanol for 1 h at room temperature. Then the cells were washed with Milli-Q water and stained with Alizarin Red.

To quantify Alizarin Red signal, the cells were washed three times with Milli-Q water and incubated in 10% acetic acid for 30 min at room temperature in a shaker. Then the collected cell suspensions were heated to 85°C for 10 min, cooled to room temperature and neutralized with 1 M sodium hydroxide (NaOH). Absorbance in resulting samples was measured at 405 nm on a plate reader (Molecular Devices, E11191). One staining was performed per well.

### Lipopolysaccharide and cyclic stretch stimulation

To establish the optimal time point for the effect of LPS on expression of genes involved in calcification (Groups 3.1 and 3.2), VICs from either healthy or calcified valves [25,000 cells per well in 24-well plates precoated with collagen I (Gibco, A10483-01)] were stimulated with 0.1 μg/ml LPS (E. coli 0111:B4, Sigma, L4391) dissolved in cell culture medium. The cells were collected after 0 (control without stimulation), 24, 48, 72, and 96 h for RNA isolation. The concentration of LPS was pre-calibrated for the most efficient induction of *ICAM1* and *BMP2* gene expression (Supplementary Figure 1).

To study gene expression after stimulation with LPS and cyclic stretch (Groups 4.1–4.8), on next day after seeding VICs were stimulated with 0.1 μg/ml LPS and/or cyclic stretch at 1 Hz (as described above) for 24 h. The experiments were performed on 125,000 VICs per well seeded on type I collagen or elastin pre-coated BioFlex 6-well cell culture plates (Dunn Labortechnik, BF-3001C and BF-3001E) and incubated at 37°C in 5% CO_2_. In the combined groups (Groups 4.4 and 4.8) the cells were stretched continuously at the same time as they were stimulated by LPS. Control samples without any treatment were maintained at the same time without mechanical stretch. The cells from control groups were cultured on BioFlex 6-well cell culture plates pre-coated with collagen I or elastin similarly to the cells from the treatment groups.

### Gene expression assay

Total RNA was isolated with Trizol reagent (Invitrogen, 15596026) according to the manufacturer's protocol, and its concentration was quantified using NanoDrop ND-1000 Spectrophotometer (Saveen Werner). Reverse transcription was performed using qScript cDNA Synthesis Kit (Quanta BioSciences Inc., 95047-500) according to the manufacturer's protocol with the following thermal cycle: 10 min at 25°C, 50 min at 42°C, and 4 min at 94°C.

Quantitative reverse transcription polymerase chain reaction (RT-qPCR) was run for markers of inflammation: intracellular adhesion molecule 1 (*ICAM1*), osteogenic differentiation: bone morphogenetic protein 2 (*BMP2*), runt-related transcription factor 2 (*RUNX2*), and periostin (*POSTN*), and myofibroblastic differentiation: α-smooth muscle actin (*ACTA2*) and calponin 1 (*CNN1*). 18S ribosomal RNA (TATAA Biocenter, qA-01-0106S) was used as the endogenous control in all experiments. PCR reactions were performed using Power SYBR Green detection PCR Master Mix (Applied Biosystems, Life technologies, 4367659). RT-qPCR was carried out in ABI7900 (Applied Biosystems) apparatus with the following amplification conditions: one cycle at 50°C for 2 min followed by 95°C for 10 min and 40 cycles of 95°C for 15 s followed by 60°C for 1 min. The fold change of gene expression compared to 18S rRNA was calculated using the comparative CT method. All PCR samples were run in duplicates. Primer sequences are presented in Supplementary Table 1.

### Statistical analysis

Principal component analysis (PCA) was performed to determine, which continuous variables discriminate between groups of cells cultured on collagen or elastin. Continuous variables were qPCR gene expression data from 2^−ΔΔ*CT*^ (RQ estimation). Markers of inflammation: *ICAM1*, osteogenic differentiation: *BMP2, RUNX2, POSTN, THBS1* (thrombospondin 1), and myofibroblastic differentiation: *ACTA2* and *CNN1* were used in PCA. PCA was performed using Phantasus tool (https://genome.ifmo.ru/phantasus/) with integrated limma instrument (Ritchie et al., 2015). Percentage contribution of each gene expression was calculated by XLSTAT-Base software.

All the other data were analyzed using GraphPad Prism 7 software (La Jolla, USA). The data in the experiment studying the effect of LPS on calcification (Groups 1.1–1.4) were analyzed using one-way ANOVA with Tukey's multiple comparisons post-test. Data are shown as mean ± SD. The timeline experiment with LPS stimulation (Groups 3.1–3.2) was analyzed using two-way ANOVA. The differences between cells from healthy and calcified valves were determined with Sidak's multiple comparison post-test. Data are shown as mean ± SEM. The data on calcification in Groups 2.1–2.5 and PCR results from Groups 4.1–4.8 showing effect of stretch and/or LPS were analyzed with one-way ANOVA or non-parametric test (Kruskal-Wallis test) because of non-equal distribution. Post-tests were done using Dunnett's multiple comparisons test (for one-way ANOVA) and Dunn's multiple comparisons test (for non-parametric test). The differences between gene expression in cells cultured on collagen or elastin were determined with paired *t*-test (for parametric data) or Wilxon matched-pairs signed rank test (for non-parametric data). Data are shown as mean ± SD. For all data Shapiro-Wilk normality test was employed to establish the type of distribution. *p* ≤ 0.05 in all data analyses was considered statistically significant.

## Results

### Induction of calcification

#### Groups 1.1–1.4

**Figure 1:**
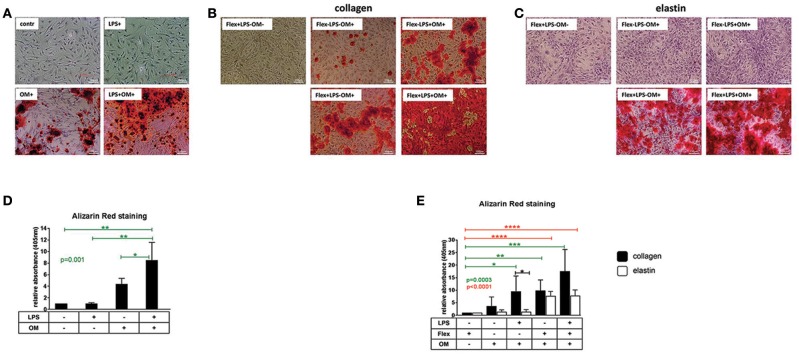
**(A)** Visualization of calcium by Alizarin Red staining of valve interstitial cells isolated from healthy aortic valves (*n* = 3) and cultured on collagen I coating for 14 days in normal control medium (contr), osteogenic medium (OM), control medium supplemented with LPS (LPS+) or osteogenic medium supplemented with LPS (LPS+OM+). × 10. **(B,C)** Visualization of calcium by Alizarin Red staining of valve interstitial cells isolated from healthy aortic valves (*n* = 6), cultured on collagen I and elastin coatings and stimulated by stretch alone in control medium (Flex+LPS-OM-), osteogenic medium alone (Flex-LPS-OM+), by LPS (Flex-LPS+OM+) and stretch (Flex+LPS-OM+), or both (Flex+LPS+OM+) in presence of osteogenic medium (OM). × 10. Experiment was performed for 14 days. Flex was applied first 7 days of experiment (2 h per day). **(D,E)** Alizarin Red staining was quantified by absorbance at 405 nm. Data were normalized to control group (LPS-OM-) **(D)** or (Flex+LPS-OM-) **(E)** equated to 1, Differences in gene expression between VICs cultured on collagen and elastin coatings are shown in black stars. The effect of stimulations on gene expression is shown in green stars for collagen and in red stars for elastin. * indicates 0.01 < *p* ≤ 0.05, ** indicates 0.001 < *p* ≤ 0.01, *** indicates 0.0001 < *p* ≤ 0.001, **** indicates *p* ≤ 0.0001. Overall *p*-values from one-way ANOVA or non-parametric analysis are shown in green for collagen and in red for elastin. Data presented as mean ± SD. *p*-values < 0.05 were considered statistically significant.

VICs from healthy aortic valves were treated with osteogenic medium for 2 weeks, resulting in robust calcification (Figures 1A,D). Stimulation of VICs with LPS alone for 2 weeks did not cause calcification, however, addition of LPS to osteogenic medium increased calcification induced by osteogenic medium alone (Figures 1A,D).

#### Groups 2.1–2.4

Stretch for 2 h per day during the first 7 days of a 2-weeks experiment alone without osteogenic medium did not cause calcification of VICs cultured on collagen and elastin coatings (Figures 1B,C,E). When the VICs were stretched in the same time frame in the presence of osteogenic medium, the calcification was augmented compared to osteogenic medium alone (Figures 1B,C,E). Combination of LPS and osteogenic medium without mechanical stretch stimulated calcification of VICs cultured on collagen, but not on elastin coating (Figures 1B,C,E).

#### Group 2.5

The combination of stretch and LPS produced an additive effect on top of that of each treatment for cells cultured on collagen, whereas cells cultured on elastin were affected only by mechanical stress (Figures 1B,C,E).

### Effects of LPS on gene expression in valve interstitial cells

#### Groups 3.1–3.2

**Figure 2:**
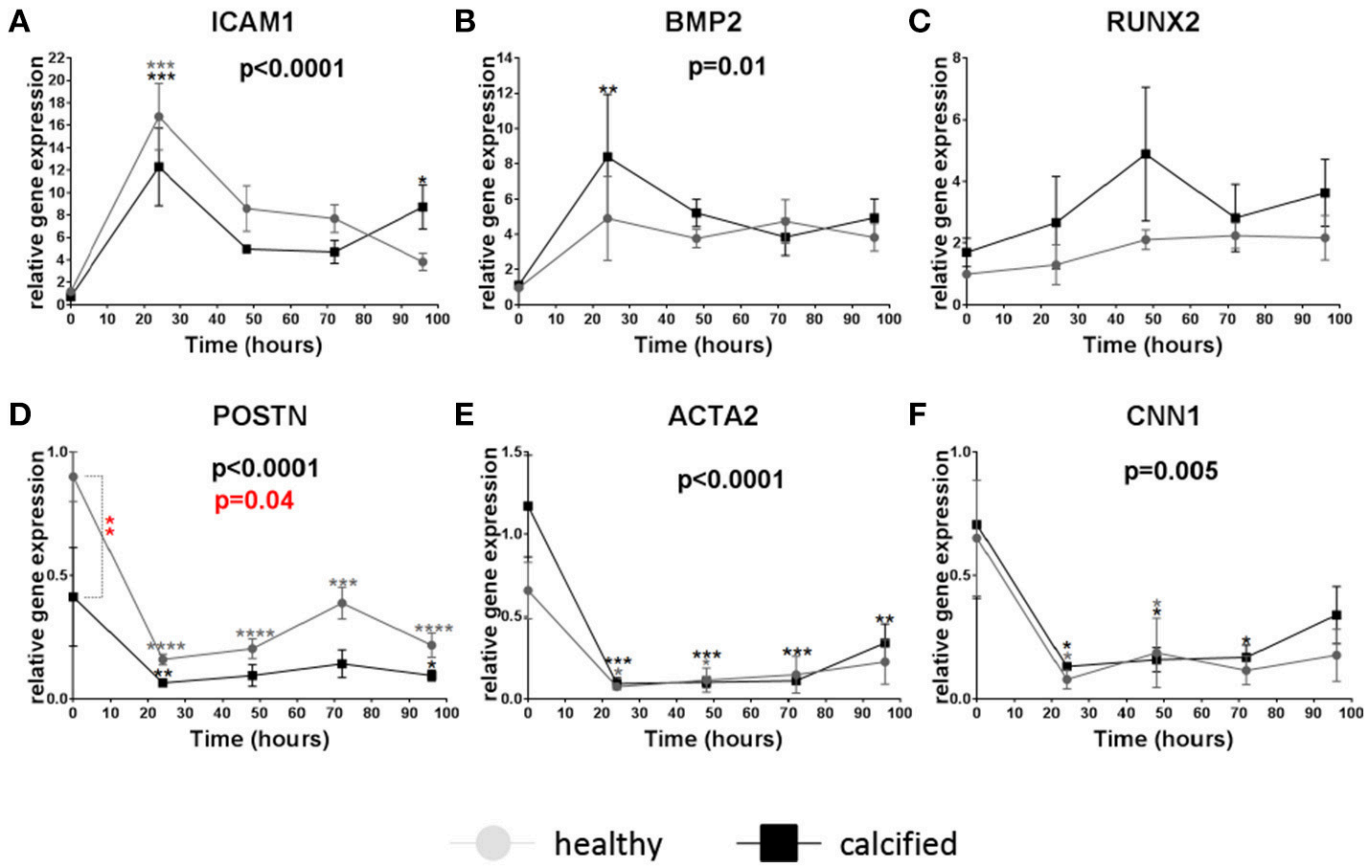
Relative gene expression of intracellular adhesion molecule 1 *(ICAM1)* **(A)**, bone morphogenetic protein 2 *(BMP2)* **(B)**, runt-related transcription factor 2 *(RUNX2)* **(C)**, periostin *(POSTN*) **(D)**, α-smooth muscle actin *(ACTA2)* **(E)**, calponin 1 *(CNN1)* **(F)** in valve interstitial cells isolated from healthy (*n* = 4, gray) and calcified (*n* = 4, black) valves cultured on collagen I coating, stimulated with LPS and collected on different time points (24, 48, 72, and 96 h). Changes in gene expression in cells with LPS stimulation in relation to control cells without LPS stimulation are shown in gray (for healthy donors) and black (for calcified donors) stars. Differences in gene expression between cells from healthy and calcified donors are shown in red stars. * indicates 0.01 < *p* ≤ 0.05, ** indicates 0.001 < *p* ≤ 0.01, *** indicates 0.0001 < *p* ≤ 0.001, **** indicates *p* ≤ 0.0001. Overall *p*-values from two-way ANOVA indicating differences in gene expression between cells from healthy and calcified donors are shown in red. Overall *p*-values from two-way ANOVA indicating effect of the time on gene expression are shown in black. Data presented as mean ± SEM. *p*-values < 0.05 were considered statistically significant.

To find the optimal time to study the effect of LPS on calcification-related genes, the time course of gene expression after 24, 48, 72, and 96 h of LPS stimulation was determined in VICs from healthy and calcified valves. *ICAM1* and *BMP2* were transiently upregulated 24 h after stimulation in cells both from healthy and calcified aortic valves without a significant difference between the two cell sources (Figures 2A,B). The expression of *RUNX2* tended to increase in cells from calcified valves after 48 h (Figure 2C). Baseline expression of *POSTN* was significantly higher in VICs from healthy valves comparing to calcified valves. After stimulation the expression of *POSTN* was inhibited in cells from both healthy and calcified valves (Figure 2D). The expression of *ACTA2* and *CNN1* was also downregulated after stimulation with LPS, but without significant differences between cells from healthy and calcified valves (Figures 2E,F).

### Gene expression in valve interstitial cells cultured on elastin or collagen

#### Groups 4.1–4.8

mRNA expression of *ICAM1, BMP2, RUNX2, POSTN, ACTA2*, and *CNN1* was measured after 24 h in VICs from calcified (*n* = 6) or healthy (*n* = 6) aortic valves stimulated with LPS and/or stretch. Cells without any stimulation were used as control. These treatments were compared in cells cultured on two different coatings: elastin and collagen mimicking the ventricular and the aortic sides of the valve leaflets. Results are presented gene-by-gene. The data presented in parentheses are mean ± SD of a ratio to control groups. See Table 2 for more details and the summary of gene expression in Groups 4.1–4.8.

**Table 2:** Summary of gene expression in Groups 4.1–4.8.

**Description of comparisons**, **normalization**	**Coating**	**Donors**	**Treatment**	**Marker of inflammation**	**Markers of osteogenic differentiation**			**Markers of myofibroblastic differentiation**		**Statistical analysis**
				***ICAM1***	***BMP2***	***RUNX2***	***POSTN***	***ACTA2***	***CNN1***
Control vs. Treatment (LPS, Flex or both) Mean of RQ values of control group (Flex-LPS-) = 1	Collagen	Healthy (*n* = 6)	Flex-LPS+	30.1 ± 22.9 *p = 0.03*	6 ± 1.6 *p = 0.9*	3.3 ± 3.4 *p = 0.9*	0.8 ± 0.5 *p = 0.99*	0.4 ± 0.4 *p = 0.03*	0.5 ± 0.2 *p = 0.001*	One-way ANOVA with Dunnett's multiple comparison post-test (for parametric data) or non-parametric test (Kruskal-Wallis test) with Dunn's multiple comparisons post-test (for non-parametric data)
			Flex+LPS-	4.8 ± 5 *p = 0.97*	1.5 ± 0.7 *p > 0.99*	2.7 ± 2.2 *p = 0.9*	1.8 ± 1.7 *p = 0.99*	0.3 ± 0.3 *p = 0.003*	0.6 ± 0.3 *p = 0.005*
			Flex+LPS+	30 ± 27.3 *p = 0.03*	33.9 ± 35.1 *p = 0.01*	11.6 ± 12.4 *p = 0.03*	1.1 ± 0.4 *p = 0.5*	0.3 ± 0.4 *p* = 0.009	0.4 ± 0.2 *p < 0.0001*
		Calcified (*n* = 6)	Flex-LPS+	41.9 ± 30.2 *p = 0.004*	5.1 ± 3.4 *p = 0.02*	2.3 ± 2.3 *p = 0.99*	0.3 ± 0.1 *p = 0.001*	0.5 ± 0.2 *p = 0.0006*	0.2 ± 0.1 *p < 0.0001*
			Flex+LPS-	11.1 ± 9.8 *p = 0.7*	2.9 ± 1.7 *p = 0.4*	0.4 ± 0.3 *p = 0.99*	0.6 ± 0.2 *p = 0.2*	0.9 ± 0.2 *p = 0.3*	1 ± 0.3 *p = 0.98*
			Flex+LPS+	31.2 ± 20.4 *p = 0.03*	7 ± 3 *p = 0.0009*	17.5 ± 19.5 *p = 0.03*	0.5 ± 0.4 *p = 0.06*	0.5 ± 0.2 *p = 0.001*	0.4 ± 0.2 *p = 0.0002*
	Elastin	Healthy (*n* = 6)	Flex-LPS+	18.7 ± 23.7 *p = 0.2*	10.8 ± 14.2 *p = 0.3*	1.3 ± 1.2 *p = 0.99*	1.4 ± 1.5 *p > 0.99*	0.8 ± 0.6 *p = 0.7*	0.8 ± 0.3 *p > 0.99*
			Flex+LPS-	0.9 ± 0.4 *p > 0.99*	7.2 ± 8 *p = 0.2*	4.9 ± 4.5 *p = 0.08*	2.1 ± 2.6 *p > 0.99*	c0.5 ± 0.2 *p = 0.03*	0.7 ± 0.5 *p = 0.2*
			Flex+LPS+	23.6 ± 19.1 *p = 0.54*	17.6 ± 13.8 *p = 0.003*	4.2 ± 3.5 *p = 0.2*	1.6 ± 1.7 *p > 0.99*	0.4 ± 0.3 *p = 0.008*	0.5 ± 0.6 *p = 0.06*
		Calcified (*n* = 6)	Flex-LPS+	5.4 ± 5.4 *p = 0.98*	0.7 ± 0.4 *p = 0.99*	0.4 ± 0.6 *p = 0.9*	0.8 ± 0.6 *p = 0.5*	0.9 ± 0.7 *p = 0.9*	0.7 ± 0.3 *p = 0.1*
			Flex+LPS-	33.6 ± 35.8 *p = 0.1*	13.4 ± 9.6 *p = 0.03*	1.4 ± 1.4 *p = 0.97*	0.7 ± 0.7 *p = 0.2*	0.3 ± 0.1 *p = 0.03*	0.5 ± 0.3 *p = 0.003*
			Flex+LPS+	39.5 ± 41.9 *p = 0.07*	17.1 ± 12.2 *p = 0.05*	3.5 ± 3.6 *p = 0.1*	1 ± 0.7 *p > 0.99*	0.4 ± 0.3 *p = 0.04*	0.3 ± 0.2 *p < 0.0001*
Collagen vs. Elastin Mean of RQ values of every treatment group of cells cultured on collagen = 1	Elastin/ Collagen	Healthy (*n* = 6)	Flex-LPS+	0.6 ± 0.9 *p = 0.51*	1.8 ± 2.4 *p > 0.9*	0.4 ± 0.3 *p = 0.3*	1.7 ± 1.8 *p = 0.8*	2.1 ± 1.5 *p = 0.03*	1.5 ± 0.6 *p = 0.03*	Paired t-test (for parametric data) or Wilcoxon matched-pairs signed rank test (for non-parametric data)
			Flex+LPS-	0.7 ± 1.3 *p > 0.99*	4.7 ± 5.2 *p = 0.09*	1.8 ± 1.7 *p = 0.3*	1.2 ± 1.5 *p = 0.7*	1.8 ± 0.9 *p = 0.06*	1.1 ± 0.9 *p > 0.99*
			Flex+LPS+	0.9 ± 0.6 *p = 0.7*	0.5 ± 0.4 *p = 0.4*	0.4 ± 0.3 *p = 0.1*	1.6 ± 1.8 *p = 0.6*	1.1 ± 0.8 *p = 0.9*	1.5 ± 1.6 *p > 0.99*
		Calcified (*n* = 6)	Flex-LPS+	0.1 ± 0.1 *p = 0.02*	0.2 ± 0.1 *p = 0.03*	0.1 ± 0.2 *p = 0.03*	3.2 ± 2.4 *p = 0.03*	1.8 ± 1.5 *p = 0.03*	3.2 ± 1.6 *p = 0.008*
							Flex+LPS-	3 ± 3.2 *p = 0.2*	4.7 ± 3.4 *p = 0.04*	3.7 ± 3.5 *p = 0.2*	1.1 ± 1.3 *p > 0.9*	0.4 ± 0.2 *p = 0.03*	0.5 ± 0.3 *p = 0.006*
			Flex+LPS+	1.1 ± 1.2 *p = 0.9*	2.5 ± 1.8 *p = 0.07*	0.2 ± 0.2 *p = 0.06*	1.9 ± 1.3 *p = 0.3*	0.7 ± 0.5 *p = 0.1*	0.6 ± 0.4 *p = 0.08*
Healthy vs. Calcified Mean of RQ values of every treatment group of cells from healthy valves = 1	Collagen	Calcified/Healthy (*n* = 6)	Flex-LPS+	1.4 ± 1 *p = 0.5*	0.8 ± 0.6 *p = 0.5*	0.9 ± 0.6 *p = 0.8*	0.3 ± 0.2 *p = 0.03*	1.3 ± 0.6 *p = 0.4*	0.4 ± 0.2 *p = 0.01*	Unpaired t-test (for parametric data) or Mann-Whitney test (for non-parametric data)
			Flex+LPS-	2.3 ± 2.1 *p = 0.2*	1.9 ± 1.1 *p = 0.1*	0.1 ± 0.09 *p = 0.05*	0.3 ± 0.1 *p = 0.1*	2.2 ± 0.4 *p = 0.09*	1.7 ± 0.6 *p = 0.05*
			Flex+LPS+	1 ± 0.7 *p > 0.99*	0.2 ± 0.1 *p = 0.09*	1.5 ± 1.7 *p = 0.5*	0.5 ± 0.4 *p = 0.07*	1.5 ± 0.5 *p = 0.2*	1.2 ± 0.5 *p = 0.5*
	Elastin		Flex-LPS+	0.3 ± 0.3 *p = 0.2*	0.1 ± 0.04 *p = 0.1*	0.3 ± 0.4 *p = 0.2*	0.6 ± 0.4 *p = 0.8*	1.1 ± 0.9 *p = 0.9*	0.9 ± 0.4 *p = 0.7*
			Flex+LPS-	10 ± 10.6 *p = 0.09*	1.9 ± 1.3 *p = 0.2*	0.3 ± 0.3 *p = 0.1*	0.3 ± 0.4 *p = 0.3*	0.7 ± 0.3 *p = 0.3*	0.8 ± 0.4 *p = 0.7*
			Flex+LPS+	1.7 ± 1.8 *p = 0.4*	1 ± 0.7 *p = 0.9*	0.8 ± 0.8 *p = 0.8*	0.6 ± 0.4 *p = 0.6*	1.1 ± 0.8 *p = 0.9*	0.5 ± 0.3 *p = 0.7*

*Relative gene expression of markers related to inflammation, osteogenic and myofibroblastic differentiation in valve interstitial cells isolated from healthy (n = 6) and calcified (n = 6) valves, cultured on collagen or elastin coatings and stimulated by LPS and stretch (“Flex”), or both. Data are normalized depending on the groups that are compared and presented as mean ± SD. Upregulated genes are highlighted in green, downregulated genes are highlighted in red. p-values < 0.05 were considered statistically significant*.

#### ICAM1

**Figure 3:**
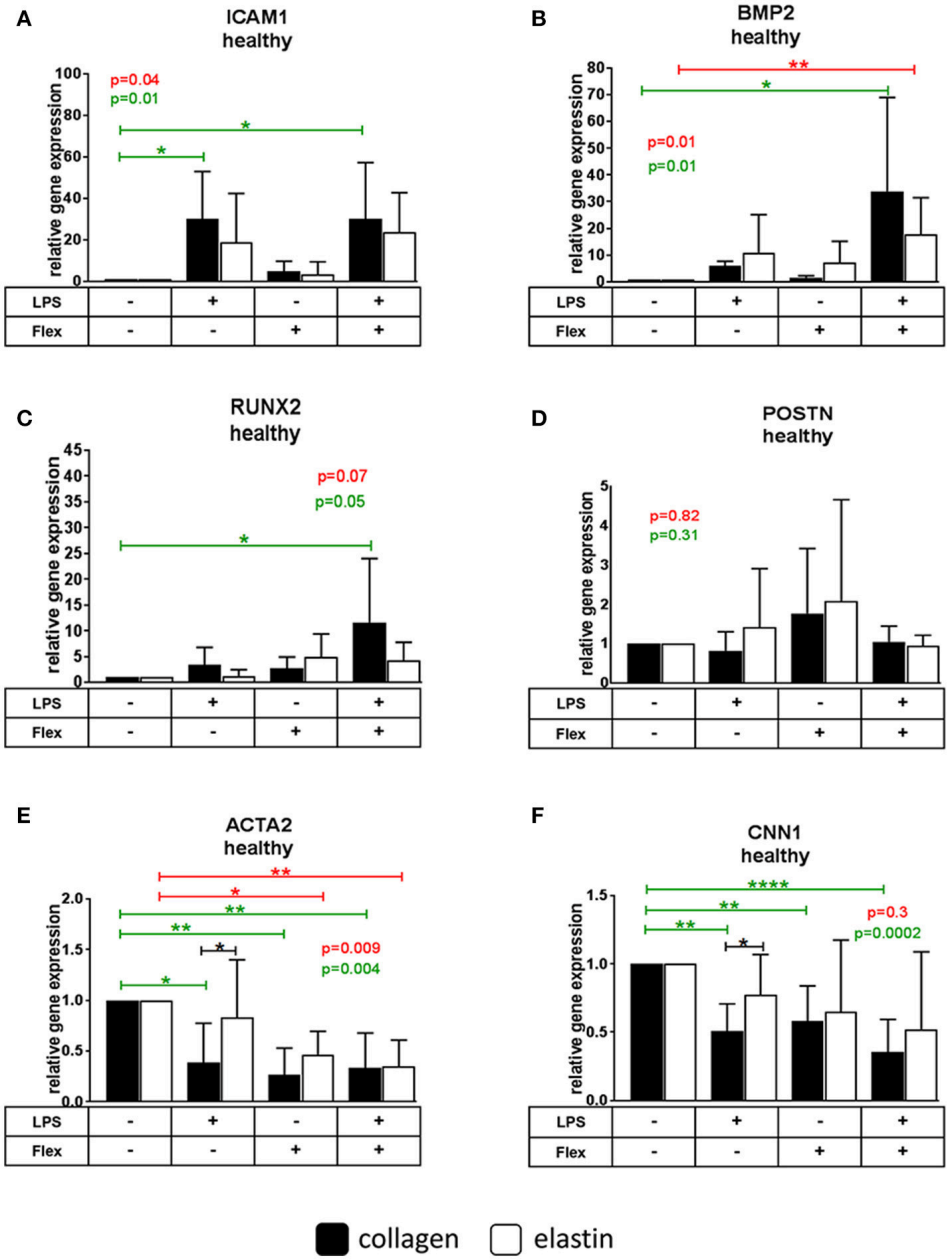
Relative gene expression of intracellular adhesion molecule 1 *(ICAM1)* **(A)**, bone morphogenetic protein 2 *(BMP2)* **(B)**, runt-related transcription factor 2 *(RUNX2)* **(C)**, periostin *(POSTN*) **(D)**, α-smooth muscle actin *(ACTA2)* **(E)**, calponin 1 *(CNN1)* **(F)** in valve interstitial cells isolated from healthy valves (*n* = 6) cultured on collagen or elastin coatings and stimulated by LPS and stretch (Flex), or both. Differences in gene expression between VICs cultured on collagen and elastin coatings are shown in black stars. The effect of stimulations on gene expression is shown in green stars for collagen and in red stars for elastin. * indicates 0.01 < *p* ≤ 0.05, ** indicates 0.001 < *p* ≤ 0.01, **** indicates *p* ≤ 0.0001. Overall *p*-values from one-way ANOVA or non-parametric analysis are shown in green for collagen and in red for elastin. All data were normalized to control group (Flex-LPS-) equated to 1. Data presented as mean ± SD. *p*-values < 0.05 were considered statistically significant.

**Figure 4:**
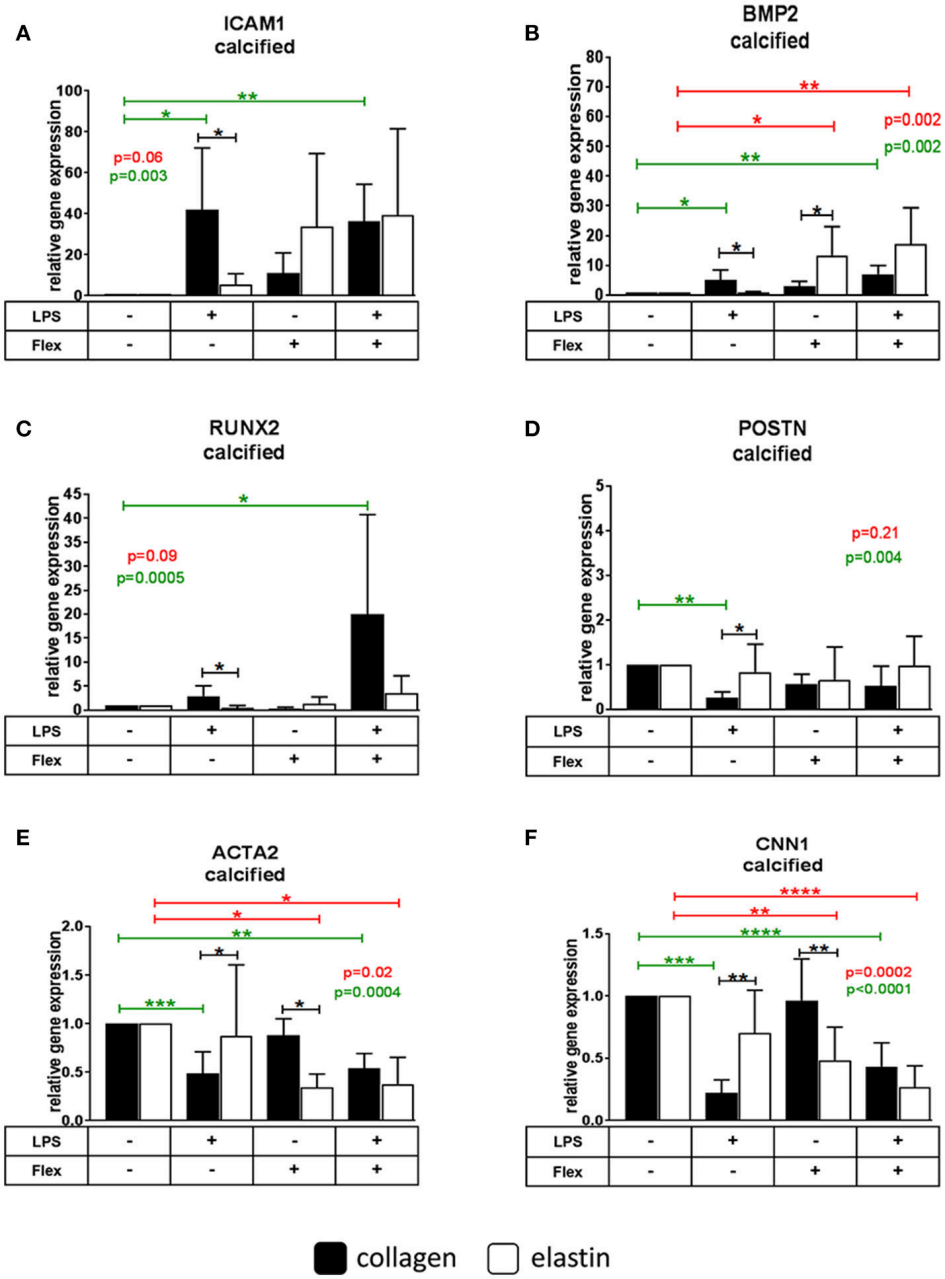
Relative gene expression of intracellular adhesion molecule 1 *(ICAM1)* **(A)**, bone morphogenetic protein 2 *(BMP2)* **(B)**, runt-related transcription factor 2 *(RUNX2)* **(C)**, periostin *(POSTN*) **(D)**, α-smooth muscle actin *(ACTA2)* **(E)**, calponin 1 *(CNN1)* **(F)** in valve interstitial cells isolated from calcified valves (*n* = 6) cultured on collagen or elastin coatings and stimulated by LPS and stretch (Flex), or both. Differences in gene expression between VICs cultured on collagen and elastin coatings are shown in black stars. The effect of stimulations on gene expression is shown in green stars for collagen and in red stars for elastin. * indicates 0.01 < *p* ≤ 0.05, ** indicates 0.001 < *p* ≤ 0.01, *** indicates 0.0001 < *p* ≤ 0.001, **** indicates *p* ≤ 0.0001. Overall *p*-values from one-way ANOVA or non-parametric analysis are shown in green for collagen and in red for elastin. All data were normalized to control group (Flex-LPS-) equated to 1. Data presented as mean ± SD. *p*-values < 0.05 were considered statistically significant.

The expression of *ICAM1* in cells from healthy and calcified valves cultured on collagen was increased after stimulation with LPS (30.1 ± 22.9 for healthy, 41.9 ± 30.2 for calcified), but not with stretch. No treatment caused significant changes in VICs cultured on elastin. *ICAM1* expression was lower in VICs from calcified, but not from healthy valves cultured on elastin (0.1 ± 0.1) compared to VICs on collagen (mean = 1) after LPS stimulation (Figures 3A, 4A).

#### BMP2

LPS stimulated *BMP2* expression in VICs from calcified (5.1 ± 3.4), but not from healthy valves on collagen. On elastin *BMP2* expression increased by stretch in cells from calcified (17.6 ± 13.8), but not from healthy valves. The combined stimulation with stretch and LPS significantly increased the expression of *BMP2* in cells from both healthy and calcified valves cultured on both collagen (33.9 ± 35.1 for healthy, 7 ± 3 for calcified) and elastin (17.6 ± 13.8 for healthy, 17.1 ± 12.2 for calcified). *BMP2* expression was lower following LPS treatment (0.2 ± 0.1) and higher following stretch (4.7 ± 3.4) on elastin compared to collagen (mean = 1) in cells from calcified, but not from healthy valves (Figures 3B, 4B).

#### RUNX2

Neither LPS nor stretch alone had significant effect on *RUNX2* expression in VICs from either calcified or healthy valves. However, combined stretch and LPS increased *RUNX2* expression in VICs from both healthy (11.6 ± 12.4) and calcified (17.5 ± 19.5) valves on collagen, but not on elastin. VICs from calcified, but not from healthy valves had lower *RUNX2* expression following LPS treatment alone on elastin (0.1 ± 0.2) compared to VICs cultured on collagen (mean = 1; Figures 3C, 4C).

#### POSTN

The expression of *POSTN* was downregulated after stimulation with LPS in VICs from calcified donors cultured on collagen (0.3 ± 0.1), but not on elastin. There were no significant differences in *POSTN* expression after stimulation with LPS and stretch in VICs from healthy valves. *POSTN* expression was higher following LPS treatment in VICs from calcified, but not from healthy valves cultured on elastin (3.2 ± 2.4) compared to collagen (mean = 1; Figures 3D, 4D).

#### ACTA2

LPS as well as the combination of stretch and LPS significantly decreased expression of *ACTA2* in VICs from healthy (0.4 ± 0.4 for LPS, 0.3 ± 0.4 for combination of stretch and LPS) and calcified (0.5 ± 0.2 for LPS, 0.5 ± 0.2 for combination of stretch and LPS) valves on collagen. In VICs from healthy valves stretch alone also decreased *ACTA2* expression (0.3 ± 0.3). *ACTA2* expression in VICs from healthy and calcified valves cultured on elastin was downregulated by stretch (0.5 ± 0.2 for healthy, 0.3 ± 0.1 for calcified) and the combination of stretch and LPS (0.4 ± 0.3 for healthy, 0.4 ± 0.3 for calcified). Expression of *ACTA2* in VICs from both healthy and calcified valves cultured on elastin was higher following LPS treatment (2.1 ± 1.5 for healthy, 1.8 ± 1.5 for calcified) compared to collagen (mean = 1; Figures 3E, 4E).

#### CNN1

The expression of *CNN1* decreased after both LPS (0.5 ± 0.2 for healthy, 0.2 ± 0.1 for calcified) and stretch (0.6 ± 0.3 for healthy) in all VICs on collagen except after stretch alone applied to VICs from calcified valves. In cells cultured on elastin the expression of *CNN1* was inhibited by stretch (0.5 ± 0.3) and the combination of stretch and LPS (0.3 ± 0.2) in VICs from calcified, but not healthy valves. The expression of *CNN1* was higher in VICs from calcified valves after stimulation with LPS (3.2 ± 1.6), but lower after stimulation by stretch (0.5 ± 0.3) on elastin compared to collagen (mean = 1; Figures 3F, 4F).

### Principal component analysis of gene expression

**Figure 5:**
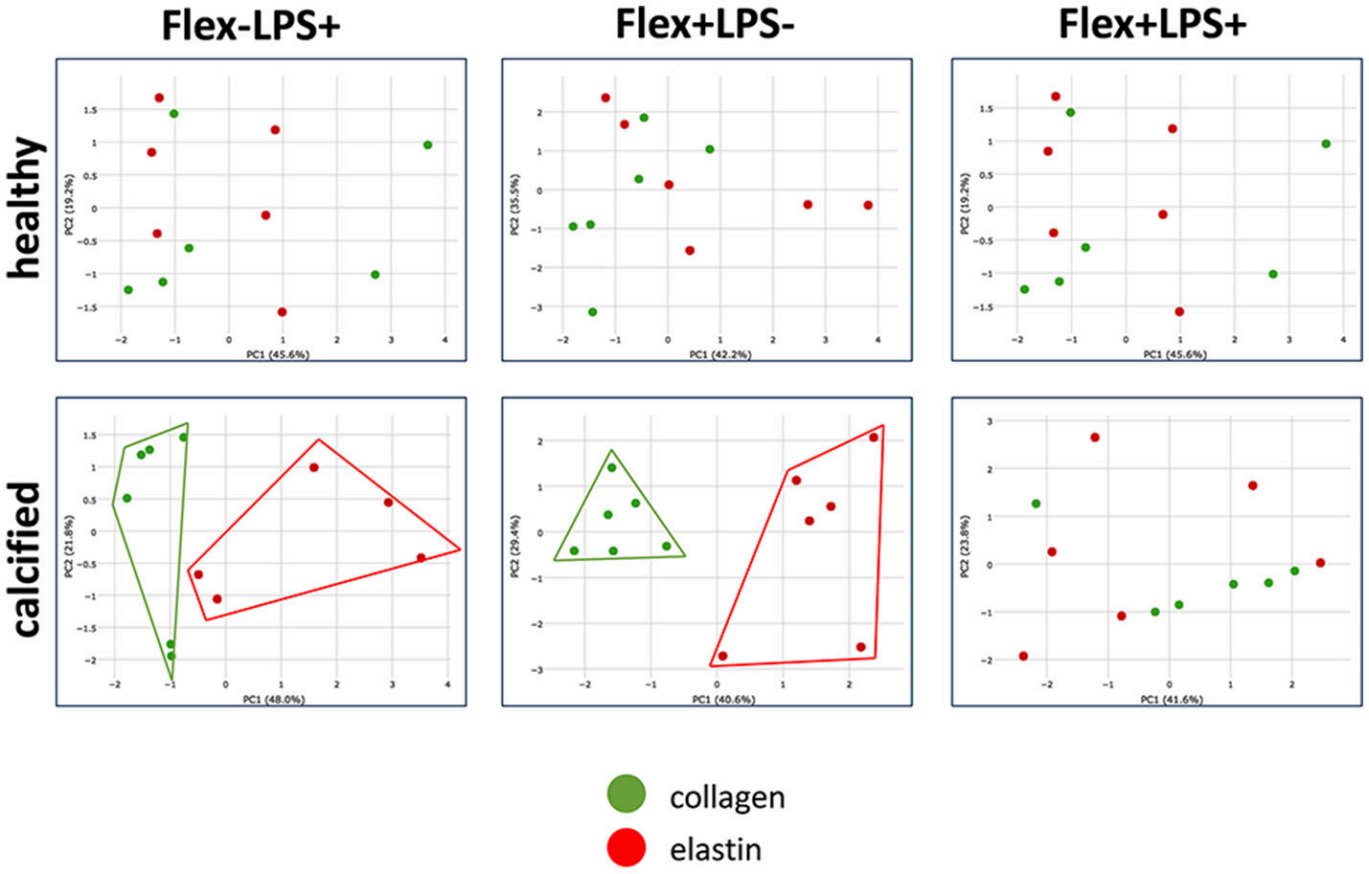
Partition of the groups based on the results of principal component analysis of gene expression in VICs cultured on collagen or elastin and stimulated by LPS, stretch or both. Principal component analysis was used to investigate does treatment with LPS or/and stretch divide VICs cultured on collagen or elastin by different groups. Expression data on a set of the following genes was used: *ICAM1, BMP2, RUNX2, POSTN, THBS1, ACTA2*, and *CNN1*. Scatter plot demonstrates that cells from calcified valves cultured on collagen (in green) and elastin (in red) form separate clusters by gene expression after simulation by LPS (Flex-LPS+) or stretch (Flex+LPS-) alone, but not in combination (Flex+LPS+). Cells from healthy valves do not form separate clusters in all cases. Each dot on scatter plot represents individual cell line (cell replicate in experiments).

To identify if cells cultured either on collagen or on elastin and stimulated with LPS, stretch or both form separate clusters by gene expression we used PCA. Each principal component (PC1 and PC2) combined expression data of all analyzed genes with different percentage contribution. Genes with highest percentage contribution in PC1 and PC2 determined cluster separation. Graphical results based on this analysis demonstrates that the cells from calcified, but not from healthy valves cultured on collagen formed a separate group from the cells cultured on elastin after stimulation with LPS and stretch alone, but not when the two treatments were combined (Figure 5). Percentage contribution of each gene expression in PCA is provided in Supplementary Table 2.

## Discussion

In the present investigation we found that mechanical stress (Flex) and inflammation (LPS) acted synergistically to stimulate expression of osteogenic genes and calcification in human VICs from healthy and calcified valves. Although inflammation and mechanical stretch had been suggested to stimulate expression of osteogenic genes (Meng et al., 2008; Lehmann et al., 2009; Yang et al., 2009a; Balachandran et al., 2010; Song et al., 2011; López et al., 2012; Ferdous et al., 2013; Wang et al., 2014) to our knowledge this is the first time a synergistic effect has been shown on osteogenic gene expression.

In the present study the VICs cultured on collagen had stronger expression of osteogenic genes after stimulation with LPS compared to VICs cultured on elastin surface. In addition, combination of LPS together with osteogenic medium stimulated calcification of cells cultured on collagen, but not on elastin. Our findings suggest the role of matrix composition in selective susceptibility of the aortic side of the valve to calcification, as it is made up predominantly of collagen while the ventricular side which is rich in elastin (Latif et al., 2005; Yip and Simmons, 2011). Our findings are in line with the previous reports stating that extracellular matrix influences ability of VICs to differentiate into osteogenic pathway (Rodriguez and Masters, 2009). Notably, not just the composition, but stiffness has a major influence (Yip et al., 2009). Surprisingly, cells cultured on elastin in our study had a stronger osteogenic response to mechanical stress. However, LPS had a more pronounced effect on collagen. Potentially, it could mean that inflammation is more crucial for calcification. Also, LPS itself may interact with matrix components: different extracellular matrix components may act as a storage, activation and delivery depots for various factors, making them more or less available to the cells (Rodriguez and Masters, 2009).

In current study we analyzed expression of a few calcification-related genes in human VICs.

*ICAM1* is an inflammatory molecule mediating pro-osteogenic gene expression in VICs in response to the LPS stimulation (Song et al., 2011; Wang et al., 2014). LPS upregulates expression of *ICAM1* on cell surface and this can down the line stimulate expression of *BMP2* (Wang et al., 2014). We found that stimulation of VICs with LPS leads to expression of *ICAM1* on collagen, but not on elastin, and it is accompanied by increased expression of *BMP2*. *BMP2* in turn activates the expression of *RUNX2* (Yang et al., 2009b), the main transcriptional regulator of osteoblastic lineage (Tkatchenko et al., 2009). In the present study, stimulation of VICs with LPS alone did not activate expression of *RUNX2*. However, stimulation of cells by LPS together with stretch upregulated *RUNX2* expression on collagen, but not on elastin. The weaker effect of LPS on *RUNX2* expression compared to *BMP2* in our 24-h time frame may reflect the notion that *RUNX2* is downstream to *BMP2* when it comes to osteogenic differentiation (Rutkovskiy et al., 2017).

The extracellular matrix protein periostin is involved in osteoblast differentiation and bone development. Knockout of *POSTN* in a murine model results in induction of several osteogenic factors including *RUNX2*, and leads to aortic valve calcification (Tkatchenko et al., 2009). The expression of *POSTN* is higher on the ventricular side of the valve and lower on the aortic side, where calcification occurs (Chen and Simmons, 2011). Our data corroborates these findings as the expression of *POSTN* was lower in VICs from calcified than from the healthy valves even without stimulation with LPS. After stimulation with LPS the expression of *POSTN* was decreased in cells cultured on collagen, but not on elastin. It is still not clear whether periostin actually contributes or opposes calcification, but most of the evidence suggests that a lack or decreased expression of periostin is pro-calcific.

Two different pathways of pathological differentiation of VICs contribute to aortic valve calcification: myofibroblastic and osteoblastic. The relative contribution of those two pathways to aortic valve calcification is unclear (Miller et al., 2011; Monzack and Masters, 2011). Myofibroblastic differentiation causes VICs to form multicellular aggregates (“nodules”) which undergo apoptosis and serve as a substrate for calcium accumulation (Miller et al., 2011). We found that upregulation of osteogenic genes (*BMP2* and *RUNX2*) by LPS and stretch coincides with downregulation of myofibroblastic markers (*ACTA2* and *CNN1*). It will be tempting to supply similar experiments with myofibroblastic differentiation. Our data provides additional evidence that myofibroblastic and osteogenic differentiation in the same cell population may be mutually exclusive (Monzack and Masters, 2011).

Our gene expression data show that the cells from calcified valves are more sensitive to LPS treatment compared to the cells from healthy valves, in line with existing studies, also showing similar differences in response to TNFα and LPS (Yu et al., 2011; Zeng et al., 2012). These data may indicate that the VICs from calcified valves already have a “pro-inflammatory” phenotype.

Calcification is the “golden” endpoint to measure osteogenic differentiation. LPS alone did not cause calcification, at least not in our timeframe and concentration. However, it enhanced the effect of osteogenic medium for cells cultured on collagen, but not on elastin coating. For analysis of calcification, mechanical stretch was applied for a short period daily. The rationale for that was a study in osteoblasts, where only short-term stimulations were sufficient to stimulate differentiation (Ignatius et al., 2005; Rutkovskiy et al., 2016), and also an effect of continuous stretch on viability and attachment of our cells in culture. In our hands mechanical stress also augmented the effect of osteogenic medium for cells cultured on collagen and elastin coatings, but did not cause calcification alone. Similar to the expression of osteogenic genes, the combination of LPS and mechanical stretch synergistically induced calcification on collagen. This suggests that heart valve calcification may be multifactorial, and different pro-calcific factors employ distinct signaling pathways.

Our study has several limitations. First, cell cultures are not fully representative of the *in vivo* situation. Second, the short time frame of our experiments does not fully represent the disease process that takes several years. Third, VICs cultured on collagen and elastin coatings were both subjected to 10% stretch by FlexCell system. However, cells on different sides of the valve and on different extracellular matrixes are exposed to different levels of mechanical stress during the cardiac cycle (Bäck et al., 2013). Our reason to subject cells to the same degree of stretch was to dissect the effect of stretch as opposed to no stretch, rather than to calibrate it to maximize or maximally diverge cellular response. Finally, the factors by which the human VICs are exposed to in the present study are only a few of the factors present in calcifying leaflets, such as ongoing matrix remodeling, invasion of immune cells, apoptosis, and others. Nevertheless, our study addresses the separate and combined effect of mechanical stretch and inflammation which is an original approach and it offers a new angle in understanding the mechanisms of aortic valve calcification.

## Conclusions

LPS-induced inflammation increases expression of osteogenic genes and inhibits expression of myofibroblastic genes in VICs cultured on collagen (representing the aortic side of the valve), but not on elastin (ventricular side of the valve). Stretch alone has modest effects on osteogenic and myofibroblastic gene expression. LPS and stretch synergistically increase calcification and osteogenic gene expression in cells cultured on collagen coating. Cells from calcified valves are more sensitive to the treatments with LPS and/or stretch than cells from healthy valves.

## Author contributions

AR conceived the idea and together with JV supervised the project. AR, JV, and MB designed the project. GS, K-OS, AM, and AnK provided critical feedback and helped with the design of the project. J-PK and M-LK organized donor collection and collected valves. MB performed experiments, collected and analyzed the data, wrote the draft of the manuscript and designed the figures. KZ and AZ helped with donor collection and cell cultures. AlK performed principal component analysis of the data. JV and AR corrected the final manuscript. All authors discussed the results and commented on the manuscript.

### Conflict of interest statement

The authors declare that the research was conducted in the absence of any commercial or financial relationships that could be construed as a potential conflict of interest.
