# Supplementary material for: Inflammation and Mechanical Stress Stimulate Osteogenic Differentiation of Human Aortic Valve Interstitial Cells
Source: Front Physiol. 2018 Nov 20;9:1635. doi: 10.3389/fphys.2018.01635 (PMC6256176; doi:10.3389/fphys.2018.01635)
Supplement: Supplementary file 1 [file Data_Sheet_1.docx]

Supplementary Material

**Inflammation and Mechanical Stress Stimulate Osteogenic Differentiation of Human Aortic Valve Interstitial Cells**

Maria Bogdanova^1*^, Aleksandra Kostina^2,3^, Katarina Zihlavnikova Enayati^1^, Arsenii Zabirnyk^1^, Anna Malashicheva^2,3,4^, Kåre-Olav Stensløkken^1^, Gareth John Sullivan^1,5,6,7^, Mari-Liis Kaljusto^8^, John-Peder Escobar Kvitting^8^, Anna Kostareva^2,12^, Jarle Vaage^9,10#^, Arkady Rutkovskiy^1,9,11#*^

^1^Department of Molecular Medicine, Institute of Basic Medical Sciences, University of Oslo, Oslo, Norway; ^2^Almazov National Medical Research Centre; ^3^ITMO University and ^4^Faculty of Biology, St. Petersburg State University, St. Petersburg, Russia; ^5^Norwegian Center for Stem Cell Research, Oslo University Hospital and University of Oslo, Oslo, Norway; ^6^Institute of Immunology, Oslo University Hospital, Oslo, Norway, ^7^Hybrid Technology Hub - Centre of Excellence, Institute of Basic Medical Sciences, University of Oslo, Oslo, Norway; ^8^Department of Cardiothoracic Surgery and ^9^Department of Emergency Medicine and Intensive Care, Oslo University Hospital, Oslo, Norway; ^10^Institute of Clinical Medicine, University of Oslo, Oslo, Norway; ^11^Department of Cardiology, Akershus University Hospital, Oslo, Norway, ^12^Department of Woman and Children Health, Karolinska Institutet, Stockholm, Sweden

^#^ Authors contributed equally to this manuscript

*****Corresponding authors:

1. Arkady Rutkovskiy, [arkady.rutkovskiy@medisin.uio.no](file:///\\lagringshotell\imb-pkfguro\01.%20Lab%20members\Aortic%20valve%20calcification%20project\PAPERS\Method%20paper\paper%20preparatiom%20for%20Life%20Science%20journal\arkady.rutkovskiy@medisin.uio.no).

2. Maria Bogdanova, [mariia.bogdanova@medisin.uio.no](mailto:mariia.bogdanova@medisin.uio.no)

**Supplementary Table 1.** Primers used for RT-qPCR.

| Gene | Description | Forward primer (5’-3’) | Reverse primer (5’-3’) |
| --- | --- | --- | --- |
| *ICAM1* | intracellular adhesion  molecule 1 | AGACAGTGACCATCTACAGCTTTCC | CACCTCGGTCCCTTCTGAGA |
| *BMP2* | bone morphogenetic  protein 2 | GCCAGCCGAGCCAACAC | CCCACTCGTTTCTGGTAGTTCTTC |
| *RUNX2* | runt-related transcription  factor 2 | GGCACTAAACAGCCTCCTCAG | GTGCTCGGATCCCAAAAGAA |
| *POSTN* | periostin | CCCAGCAGTTTTGCCCATT | TGTGGTGGCTCCCACGAT |
| *THBSP1* | thrombospondin 1 | 5'‐TCCGCAAAGTGACTGAAGAGAA‐3' | 5'‐GAACTCCGTTGTGATAGCATAGG‐3' |
| *ACTA2* | alpha -smooth muscle  actin | CCGACCGAATGCAGAAG | ACAGAGTATTTGCGCTCCGAA |
| *CNN1* | calponin | GCATGTCCTCTGCTCACTTCAA | GGGCCAGCTTGTTCTTAACCT |
| *18 S* | 18S ribosomal RNA | Catalog number: qA-01-0106S (tataabiocenter) | |

**Supplementary Table 2.** Contribution of each gene expression (%) in principal component analysis. Each principal component (PC1 and PC2) combine expression data of all analyzed genes in cells from calcified valves cultured on collagen and elastin coatings and stimulated by LPS (Flex-LPS+) or stretch (Flex+LPS-) alone, or in combination (Flex+LPS+).

|  | **Flex-LPS+** | | **Flex+LPS-** | | **Flex+LPS+** | |
| --- | --- | --- | --- | --- | --- | --- |
| **Gene** | **PC1** | **PC2** | **PC1** | **PC2** | **PC1** | **PC2** |
| *ICAM1* | 14,812 | 5,243 | 10,408 | 21,834 | 16,019 | 0,778 |
| *BMP2* | 22,281 | 12,758 | 23,068 | 0,217 | 19,849 | 2,201 |
| *SMA* | 8,872 | 27,633 | 22,790 | 0,444 | 16,721 | 2,307 |
| *RUNX2* | 12,113 | 5,089 | 7,743 | 25,220 | 26,186 | 2,400 |
| *THBS1* | 3,265 | 20,351 | 19,258 | 12,406 | 17,211 | 1,443 |
| *POSTN* | 13,234 | 26,919 | 8,642 | 7,454 | 0,039 | 62,908 |
| *CNN1* | 25,423 | 2,007 | 8,092 | 32,425 | 3,975 | 27,963 |

**
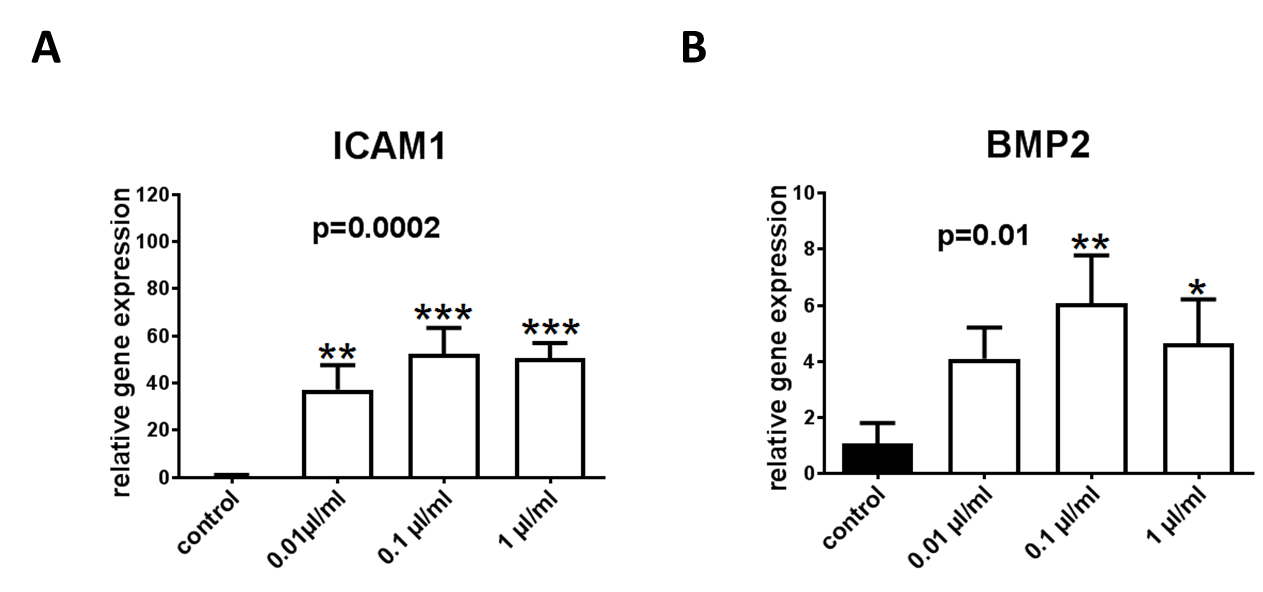
**

**Supplementary Figure 1**

Relative gene expression of intracellular adhesion molecule 1 *(ICAM1)* **(A)** and bone morphogenetic protein 2 *(BMP2)* **(B)** in valve interstitial cells isolated from healthy (n=3), valves cultured on collagen I coating and stimulated by 0.01 µl/ml, 0.1 µl/ml or 1 µl/ml LPS for 24 hours. The cells without LPS stimulation were used as control. Data were analyzed using one-way ANOVA with Dunnett’s multiple comparisons post-test.* indicates 0.01<p≤0.05, ** indicates 0.001< p≤0.01, *** indicates 0.0001< p≤0.001. Overall p-values from ANOVA is shown in bold. Data are presented as mean±SD. p-values less than 0.05 were considered statistically significant.
